# Comparison of locking plate and conservative treatment in elderly patients with displaced proximal humerus fractures

**DOI:** 10.1007/s00264-025-06425-3

**Published:** 2025-02-03

**Authors:** Uğur Bezirgan, Malik Kısmet, Yusuf Kıratlıoğlu, Mehmet Yalçın, Mehmet Armangil

**Affiliations:** Ankara University, Ankara, Turkey

**Keywords:** Conservative treatment, Elderly patients, Proximal humerus fracture, Surgical treatment

## Abstract

**Purpose:**

This study aimed to compare the outcomes of conservative treatment and locking plate osteosynthesis in displaced proximal humerus fractures in elderly patients.

**Methods:**

The study included patients over the age of 60 who were admitted to a tertiary trauma centre between 2020 and 2023, all diagnosed with 2-, 3-, or 4-part proximal humerus fractures. A total of 45 patients underwent either conservative management or locking plate fixation. In the older cohort, patients with Neer Type 2–4 fractures were treated conservatively using Velpeau immobilization. Displaced fractures, specifically 3- and 4-part fractures per the Neer classification, were treated surgically with locking plate fixation. Functional outcomes were evaluated using the Constant Shoulder score, the Disabilities of the Arm, Shoulder, and Hand (DASH) score, and the American Shoulder and Elbow Surgeons (ASES) score, with a minimum follow-up period of one year. Radiographic assessment focused on varus collapse, medial cortex displacement, greater tubercle displacement, absence of fracture lines, and callus formation. Complications, including nonunion, malunion, and avascular necrosis, were also recorded.

**Results:**

Of the 45 patients, 22 underwent locking plate fixation (Group A), while 23 were managed conservatively (Group B). In terms of fracture type, 20 patients were classified as Neer Type 2, 23 as Neer Type 3, and 2 as Neer Type 4. The mean patient age was 73.38 years. Functional scores (DASH, ASES, and Constant) were similar between the two groups, and no significant differences were observed in radiographic parameters. However, complications were significantly more frequent in the locking plate group compared to the conservative group. Two patients who underwent surgery experienced nonunion at the humeral neck. Additionally, secondary surgery was required in one patient due to postoperative infection and in another due to screw penetration into the joint. While no correlation was found between humeral neck malunions and functional outcomes, a negative correlation was observed between tubercle malunions and functional scores.

**Conclusion:**

In elderly patients with proximal humerus fractures, no significant differences in functional outcomes were observed between locking plate fixation and conservative treatment. However, locking plate fixation was associated with a higher incidence of complications and secondary surgeries. Thus, it appears that locking plate fixation does not offer superior outcomes compared to conservative management in this patient population.

## Introduction

Proximal humerus fractures are common orthopaedic injuries, especially in the elderly population. In this age group, where osteoporosis is common, even simple trauma such as falls can cause serious fractures. Proximal humerus fractures account for 6% of all fractures and are the third most common osteoporotic fracture type after wrist and hip fractures [1]. In men, 55% of these fractures are due to simple falls and the remaining 45% are due to high-energy trauma. In women, the rate of simple fall fractures increases to 82% with the effect of postmenopausal osteoporosis [2]. Displaced proximal humerus fractures are difficult to treat because of their complex anatomy and relationship to surrounding tissues [3]. Determining the optimal treatment method for such fractures is of great importance in terms of both functional and radiographic recovery.

Although traditional conservative treatment methods have advantages such as low complication rates (nonunion, malunion, and avascular necrosis (AVN)) and reduced surgical risks, they also have disadvantages such as failure to achieve adequate anatomic reduction and long healing times [4]. On the other hand, locking plate osteosynthesis is a good alternative in terms of stable fixation of fracture fragments and faster functional recovery. Over the past 14 years, there has been an increasing trend toward surgical intervention in the treatment of proximal humerus fractures in the elderly [5]. However, locking plate systems have not shown the expected success in osteoporotic bone, and complications such as varus collapse and AVN, screw perforation, deep infection, and nonunion have become common [6]. Selection of the appropriate treatment depends on the type of fracture, bone quality, deforming forces, surgeon experience and skill, patient compliance and expectations. Currently, the treatment of proximal humerus fractures, the available treatment options, and the decision-making process are still under debate [7].

The purpose of this study was to compare the clinical and radiologic outcomes of locking plate osteosynthesis and conservative treatment for displaced proximal humerus fractures in elderly patients. Functional outcomes, pain scores, complication rates, and radiographic healing processes of both treatment methods will be evaluated to determine which method is more effective in which patient population.

## Materials and methods

Informed consent was obtained from all patients for publication of all data and images. This retrospective cross-sectional study was conducted according to the tenets of the Declaration of Helsinki and approved by the Ethical Review Board (i06-479-24).

A total of 45 patients aged 60 years or older with a diagnosis of 2-, 3-, and 4-part proximal humerus fractures who were admitted to a tertiary trauma centre university hospital between December 2020 and January 2024 were included in this study.

Exclusion criteria included bipartite fractures limited to the greater or lesser tuberosity, open fractures, pathological fractures, and neurovascular injuries. Of the 76 patients initially reviewed, one with an open fracture, four with pathological fractures, and nine with Neer type 1 fractures were excluded. Additionally, two patients with fractures restricted to the tuberosity and 15 patients due to lack of follow-up and noncompliance were excluded, leaving 45 patients for the study. Patients were divided into two groups as conservative follow-up and locked plate fixation.

Routine standing anteroposterior (AP) shoulder and axillary lateral/scapular Y-radiographs and computed tomography (CT) were used to make treatment decisions. The Neer classification of proximal humerus fractures was used [8]. According to the Neer classification, the fracture was considered displaced if the fracture angulation exceeded 45° or the fracture was displaced more than 1 cm. The choice between operative and non-operative treatment was made by two experienced orthopaedic trauma surgeons together with the patient and the patient’s family. Nondisplaced 2-, 3-, or 4-part fractures (Neer 2–4) in the older age group were immobilized in velpeau for three to four weeks with the shoulder, arm in adduction and internal rotation, and elbow in flexion, and passive motion was started after two weeks. During these weeks, the patient was advised not to rest the elbow of the affected side on chairs and to sleep with a pillow behind the arm in the supine position and not to lie on the affected side. Passive and active exercises were started at the end of four. weeks and the patient was referred for standard physical therapy and rehabilitation. Mild to moderately displaced fractures with high anesthesia risk were treated conservatively.

Locked plate fixation was used for displaced neck fractures and displaced 3- and 4-part fractures. All operations were performed in the beach chair position through an anterior deltopectoral incision. Fractures were reduced under fluoroscopy and a proximal humerus locking plate was used in all surgeries.

Postoperative immobilization was performed with an arm sling for three weeks with the arm in adduction and internal rotation and the elbow in flexion. Patients were discharged approximately 3 days after surgery and sutures were removed after 21 days. Passive and active exercises were started at three weeks postoperatively, regardless of osteosynthesis stability and bone quality. Patients were then referred for physical therapy and rehabilitation.

Forty-five patients admitted to our hospital between December 2020 and January 2024 were retrospectively reviewed with follow-up periods of three, six, nine and 12 months from the date of admission. The functional outcomes of the patients at approximately three months, six months, and one year were evaluated using the Constant Shoulder Score, The disabilities of the arm, shoulder and hand (DASH) questionnaire, and American Shoulder and Elbow Surgeons Shoulder Scores (ASES). The Constant score [9] was used to assess shoulder pain (range, 0 to 15 points), strength (range, 0 to 25 points), activities of daily living (range, 0 to 20 points), and range of motion (range, 0 to 20 points). The DASH [10] is a 30-item self-report questionnaire with response options presented as 5-point Likert scales. Scores range from 0 (no disability) to 100 (most severe disability). The ASES score [11] is a 10-item measure of shoulder pain and function. Pain is rated on a 10 cm visual analog scale (VAS) and accounts for 50% of the total score. The remaining 50% of the score is determined by responses to 4-point Likert scale questions about physical function.

**Fig. 1:**
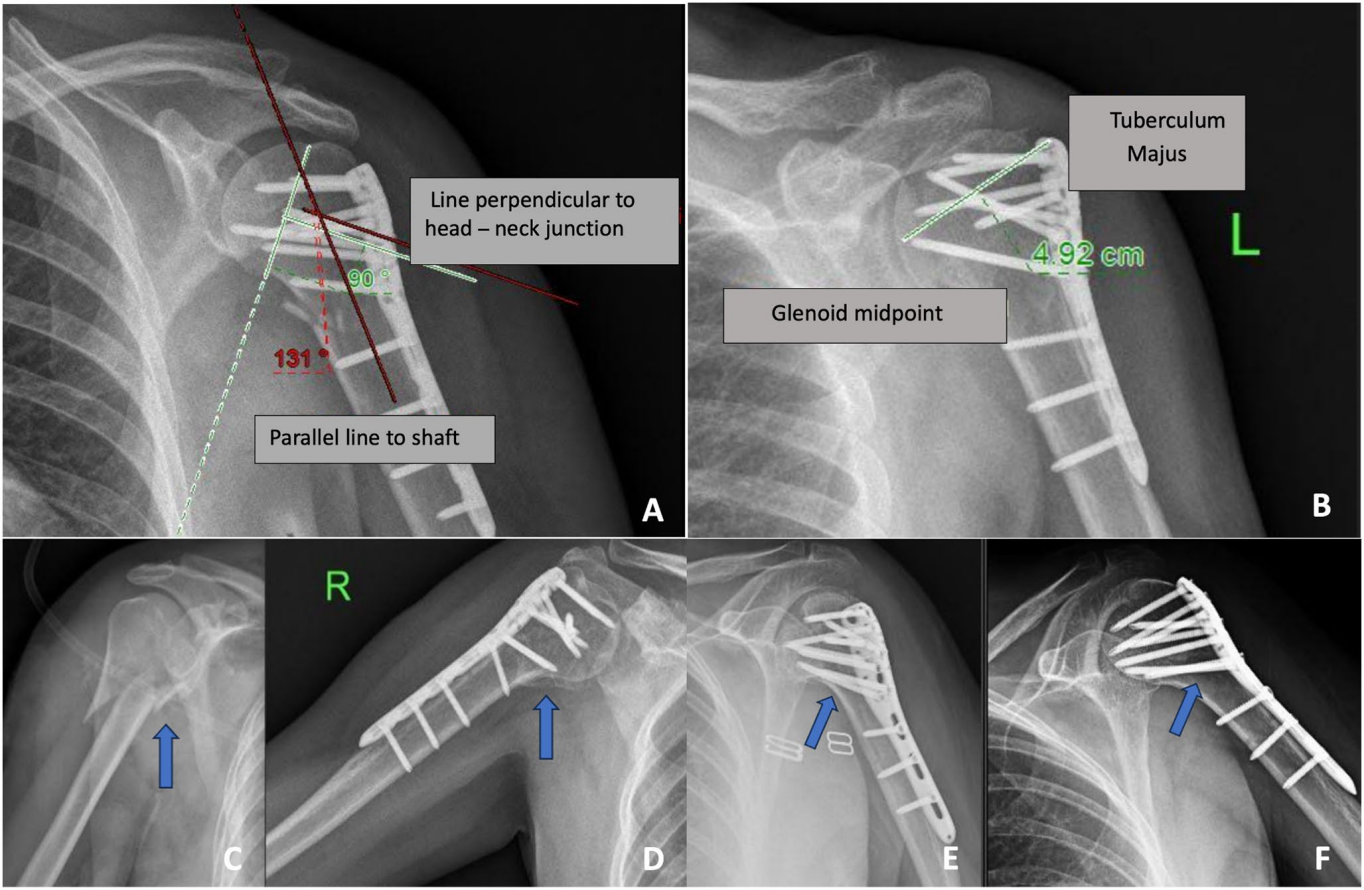
**A**: Head and neck angle calculation. **B**: Distance between tuberculum majus and glenoid. **C**-**D**: Anatomic reduction of medial cortex preoperative and postoperative. **E**-**F**: Inferomedial locking screw

Radiographic evaluations were based on the following criteria: head-neck angle (varus collapse) (Fig. 1-A), medial cortical displacement (medial column instability), greater tubercle-glenoid distance (greater tubercle displacement) (Fig. 1-B), absence of fracture lines and callus formation (healing). Radiographs in the anteroposterior shoulder and axillary lateral/scapular Y views were used to assess fracture healing, neck-shaft angle [12], and presence of medial support [13]. The presence of medial support was defined as anatomical reduction of the medial cortex (Fig. 1-C-D) and an inferomedially positioned oblique locking screw (Fig. 1- E-F). Complications included nonunion, malunion, and AVN.

### Statistical analysis

Statistical analysis of obtained data was performed using IBM PSAW 18 software (SPSS Inc, Chicago, IL, USA). To compare continuous outcomes variables between the locking plate and nonsurgical treatment groups, independent sample t tests were used when data was normally distributed and the Mann-Whitney U test for non-parametric metric data. Chi-squared test was used for categorical data. A value of P 0.05 was considered significant. Pearson correlation coefficients were calculated to measure correlations.

## Results

Of the 45 patients included in the study, 22 patients were treated with locking plate (group A) and 23 patients were treated conservatively (group B). Twenty patients were classified as Neer type 2, 23 as Neer type 3, and 2 as Neer type 4 fractures. The mean age of the patients was 73.38 years (minimum 60 - maximum 91 years).

In the functional evaluation, DASH, ASES, and Constant shoulder scores were similar between the two groups. There was no significant difference in radiographic parameters between the two groups. However, significant differences in complications were observed in the locking plate group compared to the conservative group. Malunion was observed in one patient who underwent surgery and in three patients who were treated conservatively. Nonunion of the humeral neck was observed in two patients who underwent surgery. One patient who underwent locking plate surgery developed postoperative infection and underwent secondary surgery. In addition, another patient required secondary surgery due to screw perforation.

While there was no correlation between humeral neck malunions and functional scores, a negative correlation was observed between tubercle malunions and functional scores. This suggests that tubercle malunions have a negative impact on functional outcomes. Patient-based assessment results were summarized in Table 1.

**Table 1:** Patient-based assessments of age, Neer classification type, functional status, radiologic data and complications

	Total *N* = 45	Surgical Treatment Group *N* = 22	Conservative Treatment Group *N* = 23	*p*
**AGE** (mean ± SD)	73.38 ± 12.97	72.77 ± 11.72	74.91 ± 12.31	0.4230
**Neer Classification** N(%)				**0.011**
2 3 4	20 (44.44) 23 (51.11) 2 (4.44)	5 (22.73) 15 (68.18) 2 (9.09)	15 (65.22) 8 (34.78) -	
**Functional status** (mean±SD)				
VAS DASH score ASES score Constant score	3.6 ± 2.61 31.25 ± 32.57 67.13 ± 26.42 64.51 ± 25.61	4.04 ± 3.04 25.63 ± 30.54 65.33 ± 29.34 61.27 ± 29.41	3.17 ± 2.1 36.62 ± 34.19 68.84 ± 23.83 67.61 ± 21.58	0.231 0.217 0.649 0.413
**Radiological data** (mean ± SD), N(%)				
Head-neck angle Medial cortical absence T. majus – glenoid distance(cm) Fractur line Callus	134.64 ± 11.39 30 (66.67) 4.80 ± 0.52 25 (55.56) 23 (51.11)	133.95 ± 9.52 13 (59.09) 4.65 ± 0.40 11 (50.00) 7 (31.82)	136.17 ± 12.95 17 (73.91) 4.94 ± 0.60 14 (60.87) 16 (69.57)	0.363 0.292 0.062 0.463 **0.011**
**Complications**				
Malunion Nonunion AVN Implant related	4 (8.89) 2 (4.44) 1 (2.22) 3 (6.67)	1 (4.55) 2 (9.09) 1 (4.55) 3 (13.64)	3 (13.04) - - -	0.223 0.301 0.067

VAS: Visual Analog Scale, DASH: The disabilities of the arm, shoulder and hand, ASES: American Shoulder and Elbow Surgeons Shoulder, AVN: Avascular Necrosis, SD: Standard deviation

In this study, patients treated conservatively showed similar improvement in range of motion (ROM) in all planes of motion compared to patients treated with plate fixation. However, surgical patients had a shorter time to functional ROM. Detailed joint ROMs are shown in the Table 2.

**Table 2:** Post-treatment ROM of flexion, external rotation and abduction

		Surgical Treatment Group *N* = 22	Conservative Treatment Group *N* = 23	*p*
**Flexion**	3 months	141.20 ± 4.53	121.54 ± 10.83	**0.000**
	6 months	146.69 ± 3.09	127.16 ± 9.68	**0.000**
	12 months	150.95 ± 2.60	147.06 ± 7.61	0.059
	p*	**0.0000**	**0.0000**	
**External rotation**	3 months	27.68 ± 2.21	17.99 ± 3.84	**0.000**
	6 months	31.84 ± 1.67	23.06 ± 4.05	**0.000**
	12 months	33.48 ± 1.51	32.64 ± 2.97	0.285
	p*	**0.0000**	**0.0000**	
**Abduction**	3 months	125.68 ± 7.83	123.03 ± 8.40	0.228
	6 months	130.01 ± 8.30	128.19 ± 7.97	0.363
	12 months	137.79 ± 9.12	140.06 ± 9.02	0.406
	p*	**0.0000**	**0.0000**	

*This p-value is for the Friedman test performed on the 3rd, 6th, and 12th-month values. Post-hoc analysis showed that the difference was significant for both 3rd-6th and 6th-12th months for all measurements in both groups

## Discussion

In this study comparing internal fixation with locking plate and conservative treatment of displaced proximal humerus fractures in the elderly, there was no significant difference in functional and radiologic outcomes, but the complication rate was higher in the locking plate group.

**Fig. 2:**
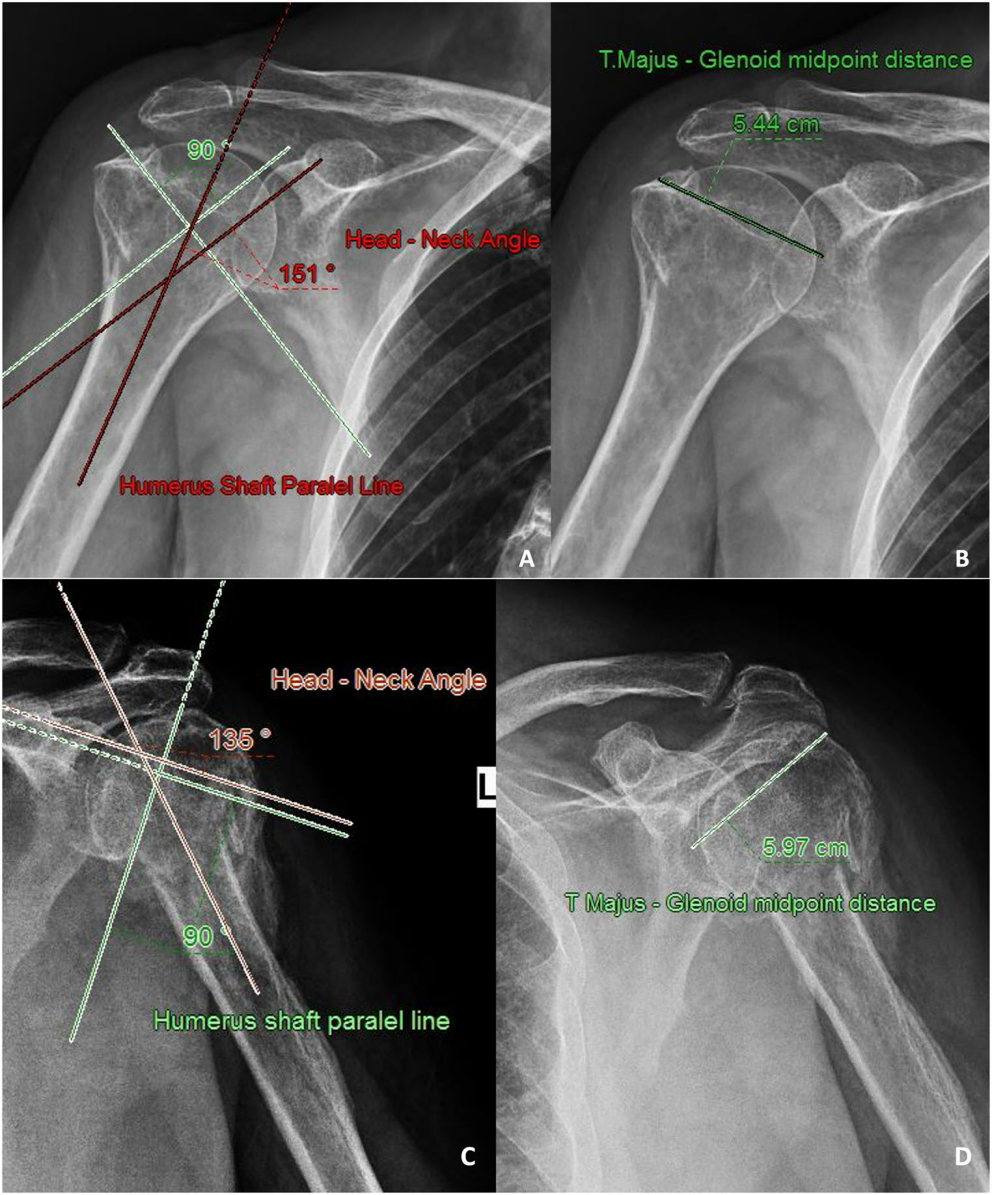
One-year radiological images of two patients with conservatively treated proximal humerus fractures. (**A**-**B**) Patient 1, (**C**-**D**) Patient 2. (**A**, **C**) Measurements of the head-neck angle; (**B**, **D**) Measurements of the greater tuberosity–glenoid distance

The DASH, ASES, and Constant shoulder scores used for functional assessment were similar between the two groups, indicating that both treatment methods were effective in improving patients’ shoulder function. There was no significant difference in radiographic parameters between the two groups (Fig. 2). These results suggest that there is no significant difference between conservative and surgical treatment in terms of functional and anatomical improvement in the short and medium term.

However, the potential risks of surgical treatment must be considered when evaluating these results. Surgical interventions carry certain risks, particularly in elderly patients and those with comorbidities. When planning surgery for proximal humerus fractures in elderly patients, a multidisciplinary approach should be used to minimize anaesthesia-related risks. Anesthesiologists, surgeons, and geriatricians should work together to evaluate the patient’s overall health status, existing comorbidities, and potential benefits of surgical intervention [14].

Regional anesthesia may reduce the risk of cardiovascular and respiratory complications compared with general anaesthesia. Techniques such as interscalene block may provide effective pain control and reduce the need for systemic anesthesia in proximal humerus fracture surgery [15].

Considering that the patients in the study were over 60 years of age, their possible comorbidities, and the risk of a second surgery, it is clear that there is no real benefit in plate fixation according to the residual functional demand of the affected limb.

Medial fragmentation, varus angulation, and calcar restoration should be considered when choosing the type of treatment (conservative vs. surgical) [16]. In addition, the patient’s age and functional expectation, possible comorbidities, and the degree of fracture displacement are other criteria used to make the decision for surgery [17]. Although there is currently no clear indication for surgical treatment of proximal humeral fractures, it is believed that surgery may be preferred in young patients with unstable fractures and significant fragment displacement [18].

Mathur et al. analyzed the correlation between radiological outcomes and functional results in the surgical treatment of proximal humerus fractures using locked plates. Their findings indicated that a higher neck-shaft angle, greater head-shaft angle, larger head diameter and height, superior positioning of the greater tuberosity relative to the articular surface, and proper reduction of the medial hinge were all associated with improved functional outcomes [19].

To restore proper function of the shoulder joint, optimal and correct positioning of the rotator cuff and the greater and lesser tubercles of the humerus is of paramount importance. Proper alignment of these anatomical structures ensures smooth and pain-free shoulder motion. In particular, the function of the supraspinatus and infraspinatus muscles depends on the correct position of the greater tubercle [20].

**Fig. 3:**
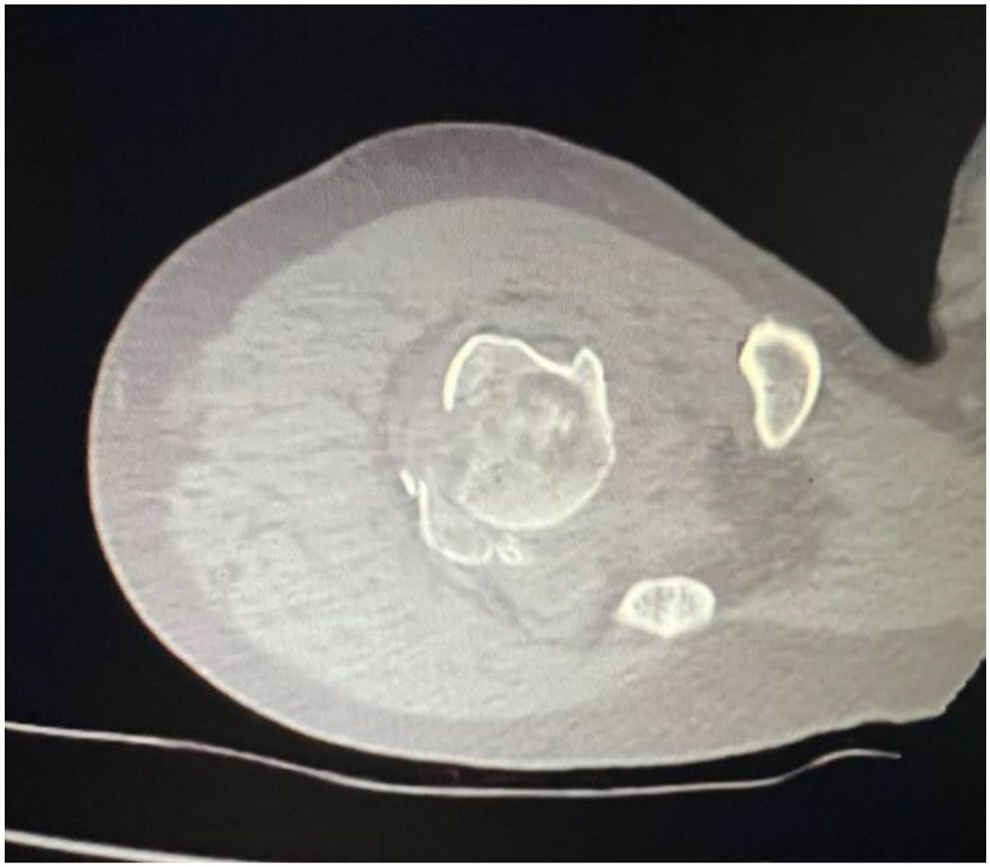
Tuberculum majus displaced fracture

The most common posterosuperior displacement of the greater tubercle in the subacromial space results in impaired shoulder function. Such displacements (> 5 mm) can prevent proper function of the rotator cuff muscles, which can lead to limited shoulder motion and pain [21]. In conclusion, correct restoration of the anatomical structure in the treatment of shoulder fractures plays a critical role in improving the functional outcome of patients. In this study, tubercle malunion was associated with poor functional outcome. Based on this study, displaced tuberculum majus fractures can be considered as the only type of proximal humerus fracture that can be treated conservatively with poor outcome (Fig. 3).

**Fig. 4:**
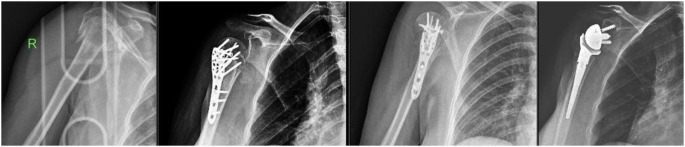
Avascular necrosis and reverse shoulder arthroplasty in the postoperative follow-up period

One of the greatest challenges in the management of proximal humerus fractures is the complexity of the fracture and its effect on the vascularization of the humeral head. Available data on three- and four-part fractures indicate the possibility of vascular injury, dislocation and cartilage damage of the humeral head, with necrosis and collapse of the head in 30–100% of cases [18]. This may adversely affect the healing process by preventing adequate blood supply to the bone and soft tissues at the fracture site. In this study, the surgical neck fracture did not heal in two patients, one of whom developed AVN and was reversed with a reverse shoulder prosthesis (Fig. 4).

**Fig. 5:**
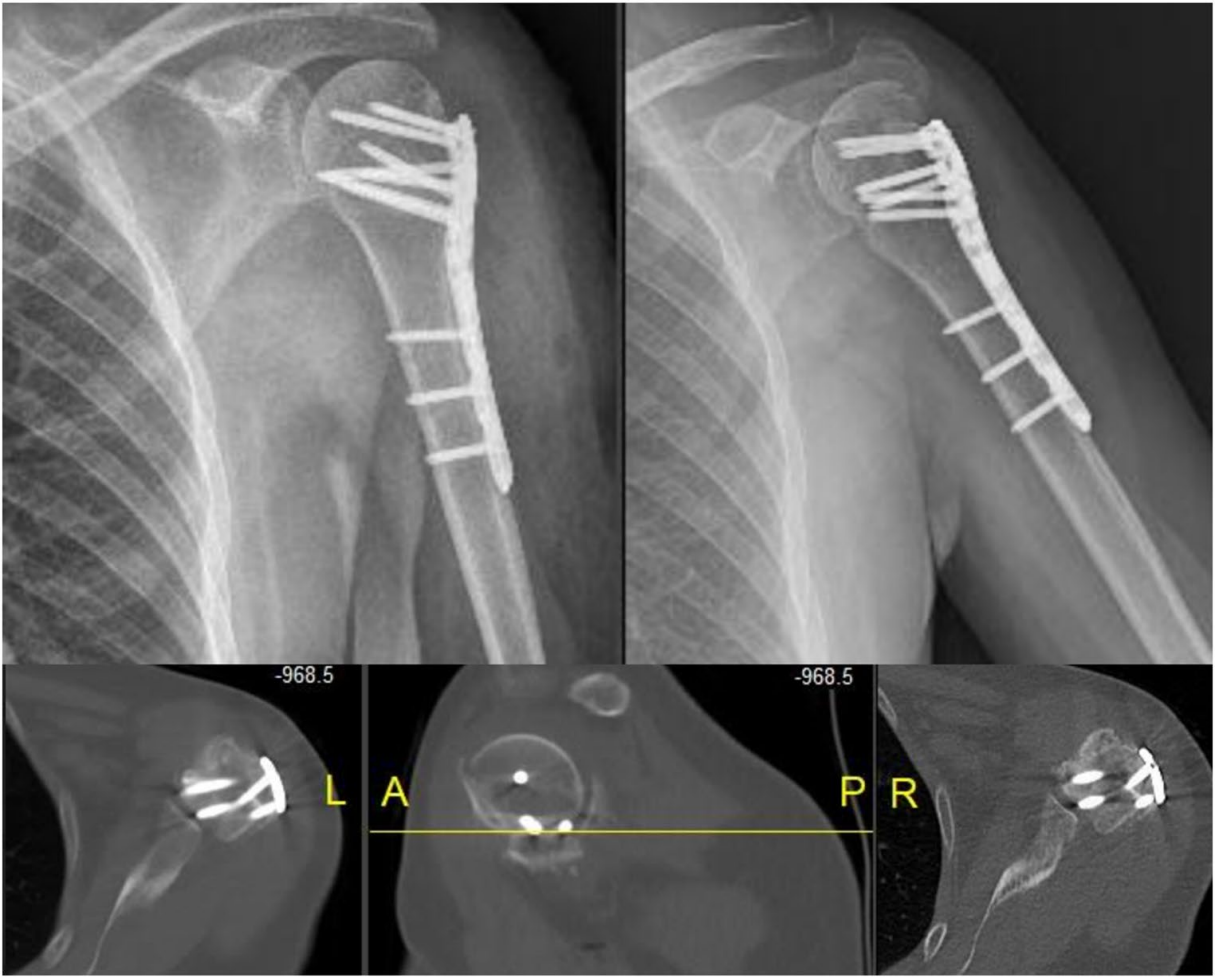
X-ray and CT Imaging of the screw as it extends into the glenoid cavity The screws were revised due to their excessive length and encroachment into the joint line

In a study by Kavuri et al. on complications of locking plate fixation, 13.8% required revision surgery [6]. Similarly, in our study, in two patients who underwent surgery, nonunion of the humeral neck and screw joint perforation in one patient required secondary surgery (Fig. 5). In some studies in the literature, the results of plate fixation were similar to ours compared with conservative treatment, but the complication rate was higher in patients in the surgical group [22–24]. In addition, unlike in our study, healing of the surgical neck in varus or valgus did not change the outcome in the conservatively treated patients.

**Fig. 6:**
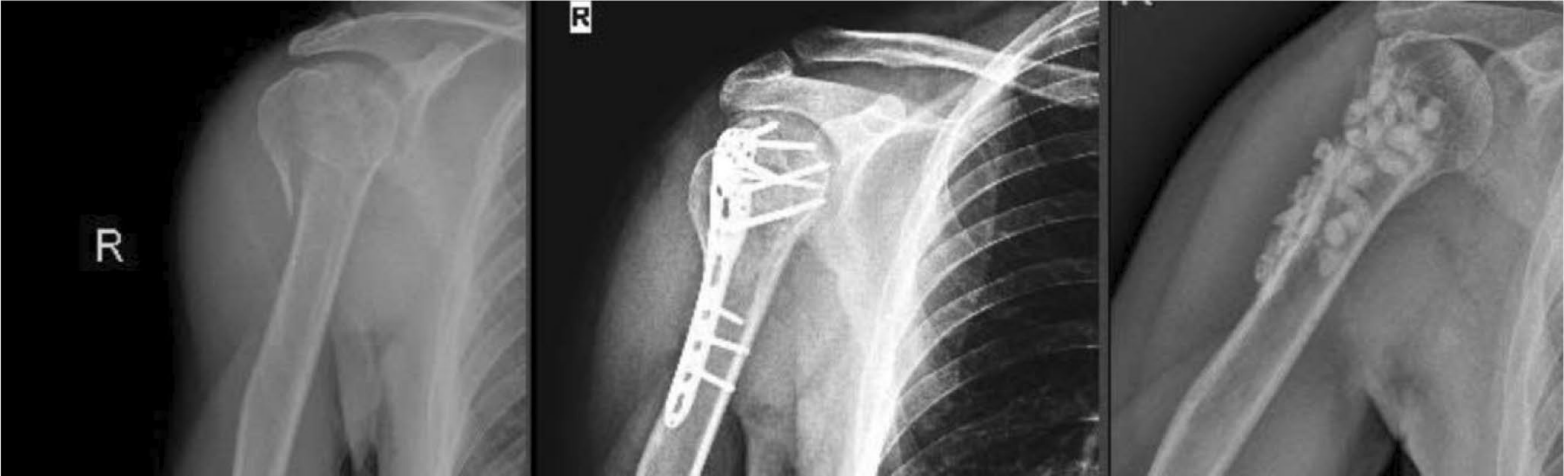
Post-operative infection, implant removal and antibiotic bead application

Among the complications observed in patients with locking plates, cases of deep infection are also noteworthy. Deep infection is defined as osteomyelitis, which is a deep-seated infection in the bone accompanied by chronic suppuration and release of microbiological agents. Such an infection is usually caused by the bacterium Staphylococcus aureus. Treatment of such infections is usually successfully accomplished by lavage (cleaning and washing) of the infected area. In our study, postoperative infection was observed in one patient who underwent locking plate placement and required surgical intervention (Fig. 6). In this case, the pathogen was S. aures and antibiotic treatment was required for 2.5 weeks. In this case, bead application of antibiotic bone cement in addition to irrigation was important to prevent the infection from becoming deep and chronic.

The retrospective design of the study, the small number of patients enrolled in the study, and the lack of comparison with other surgical treatments are some limitations.

According to the data obtained from our experience in this study, conservative treatment seems to be a very good alternative to surgical option in proximal humerus fractures, especially in patients over 60 years of age with comminuted fractures. Considering the high incidence and high cost of proximal humerus fractures, there is currently no valid scientific evidence regarding the best treatment method [25]. The choice of conservative or surgical treatment depends on the experience and practice of the surgeon after evaluation of the patient’s general clinical condition and fracture pattern. Although the indications for surgery in the treatment of proximal humerus fractures are gradually narrowing, significant results can be achieved with good surgery in well-selected cases.

## Conclusion

In elderly patients with proximal humerus fractures, no significant differences in functional outcomes were observed between locking plate fixation and conservative treatment. However, locking plate fixation was associated with a higher incidence of complications and secondary surgeries. Thus, it appears that locking plate fixation does not offer superior outcomes compared to conservative management in this patient population.

## Data Availability

No datasets were generated or analysed during the current study.
